# *Plantago ovata* Mucilage in the Design of Fast Disintegrating Tablets

**DOI:** 10.4103/0250-474X.51952

**Published:** 2009

**Authors:** S. B. Shirsand, Sarasija Suresh, M. S. Para, P. V. Swamy, D. Nagendra Kumar

**Affiliations:** Department of Pharmaceutical Technology, H.K.E. Society's College of Pharmacy, Sedam Road, Gulbarga-585 105, India; 1 Department of Pharmaceutics, Al-Ameen College of Pharmacy, Near Lal Bagh Main Gate, Hosur Road, Bangalore-560 027, India; 2 Department of Pharmaceutics, S. V. E. T's College of Pharmacy, Humnabad-585 330, India

**Keywords:** Prochlorperazine maleate, *Plantago ovata* mucilage, fast-disintegrating tablets, crospovidone

## Abstract

In the present work, fast disintegrating tablets of prochlorperazine maleate were designed with a view to enhance patient compliance by direct compression method. In this method mucilage of *Plantago ovata* and crospovidone were used as superdisintegrants (2-8% w/w) along with microcrystalline cellulose (20-60% w/w) and directly compressible mannitol (Pearlitol SD 200) to enhance mouth feel. The prepared batches of tablets were evaluated for hardness, friability, drug content uniformity, wetting time, water absorption ratio and *in vitro* dispersion time. Based on *in vitro* dispersion time (approximately 8 s), the two formulations were tested for the *in vitro* drug release pattern (in pH 6.8 phosphate buffer), short-term stability (at 40°/75% relative humidity for 3 mo) and drug-excipient interaction (IR spectroscopy). Among the two promising formulations, the formulation prepared by using 8% w/w of *Plantago ovata* mucilage and 60% w/w of microcrystalline cellulose emerged as the overall best formulation (t_50%_ 3.3 min) based on the *in vitro* drug release characteristics compared to conventional commercial tablets formulation (t_50%_ 17.4 min). Short-term stability studies on the formulations indicated that there are no significant changes in drug content and *in vitro* dispersion time (p<0.05).

Mucilage is most commonly used as adjuvant in the manufacturing of different pharmaceutical dosage forms. They possess a variety of pharmaceutical properties, which include binding, disintegrating, suspending, emulsifying and sustaining properties at different proportion in different pharmaceutical dosage forms[1–4]. Natural mucilages are preferred over semi-synthetic and synthetic materials due to their non-toxic, low cost, free availability, emollient and non-irritating nature[5,6]. Ispaghula mucilage consists of epidermis of the dried seeds of *Plantago ovata*. The present work was carried out to study the disintegrant property of *Plantago ovata* mucilage in comparison with crospovidone by formulating fast disintegrating tablets of prochlorperazine maleate.

Many patients express difficulty in swallowing tablets and hard gelatin capsules, tending to non-compliance and ineffective therapy[7]. Recent advances in novel drug delivery systems (NDDS) aim to enhance safety and efficacy of drug molecule by formulating a convenient dosage form for administration and to achieve better patient compliance. One such approach is fast disintegrating tablets[7–10]. Prochlorperazine maleate (PCZM) is a phenothiazine antipsychotic and widely used in prevention and treatment of nausea, vomiting including that associated with migraine or drug-induced emesis[11]. The concept of formulating fast disintegrating tablets containing prochlorperazine maleate offers a suitable and practical approach in serving desired objective of faster disintegration and dissolution characteristics with potential increased bioavailability.

## MATERIALS AND METHODS

Prochlorperazine maleate was a gift sample from Mehta Pharmaceuticals, Mumbai. Crospovidone was gift sample from Wockhardt Research Centre, Aurangabad. Directly compressible mannitol (Pearlitol SD 200), microcrystalline cellulose (MCC, PH-102) and sodium stearyl fumarate (SSF), all were obtained as generous gifts from Strides Arcolabs, Bangalore. All the other chemicals were of analytical grade.

### Isolation of mucilage:

For the isolation of mucilage[6,12], seeds of *Plantago ovata* were used. They were soaked in distilled water for 48 h and then boiled for 1 h for complete release of mucilage into water. The material was filtered by squeezing in a muslin cloth to remove marc. Then equal volume of acetone was added to filtrate to precipitate the mucilage. The mucilage was separated and dried in oven at a temperature less than 60°, powdered (#60 mesh), weighed and stored in desiccator until further use.

### Preparation of fast disintegrating tablets of prochlorperazine maleate:

Fast disintegrating tablets of prochlorperazine maleate were prepared by direct compression method[13] according to the formulae given in Table 1. All the ingredients were passed through #60 mesh separately. The drug and MCC were mixed by taking small portion of both each time and blending it to get a uniform mixture and kept aside. Then the other ingredients were weighed and mixed in geometrical order and tablets were compressed using 7 mm round flat punches to get tablets of 150 mg weight on a 10-station rotary tablet machine (Clit, Ahmedabad). A batch of 60 tablets was prepared for all the designed formulations.

**TABLE 1:** COMPOSITION OF DIFFERENT BATCHES OF FAST DISINTEGRATING TABLETS OF PROCHLORPERAZINE MALEATE

Ingredients (mg/tablet)	Formulation code								
	
	DC_0_	DCP_1_	DCP_2_	DCP_3_	DCP_4_	DPM_1_	DPM_2_	DPM_3_	DPM_4_
Prochlorperazine maleate	5.0	5.0	5.0	5.0	5.0	5.0	5.0	5.0	5.0
Cross-povidone	--	3.0	3.0	6.0	12.0	--	--	--	--
*Plantago ovata* mucilage	--	--	--	--	--	3.0	3.0	6.0	12.0
Microcrystalline cellulose	--	--	30.0	60.0	90.0	--	30.0	60.0	90.0
Aspartame	3.0	3.0	3.0	3.0	3.0	3.0	3.0	3.0	3.0
Sod stearyl fumarate	1.5	1.5	1.5	1.5	1.5	1.5	1.5	1.5	1.5
Talc	3.0	3.0	3.0	3.0	3.0	3.0	3.0	3.0	3.0
Pine apple flavour	1.5	1.5	1.5	1.5	1.5	1.5	1.5	1.5	1.5
Mannitol qs (Pearlitol SD 200)	136.0	133.0	103.0	70.0	34.0	133.0	103.0	70.0	34.0

DC_0_= Control formulation without superdisintegrant, DCP = Formulation containing crospovidone as a superdisintegrant, DPM = Formulation containing *Plantago ovata* mucilage as superdisintegrant.

### Evaluation of tablets:

Twenty tablets were selected at random and weighed individually. The individual weights were compared with the average weight for determination of weight variation[14]. Hardness and friability of the tablets were determined by using Monsanto Hardness Tester and Roche friabilator, respectively. For content uniformity test, ten tablets were weighed and powdered. The powder equivalent to 5 mg of PCZM was extracted into methanol and liquid was filtered (Whatman No. 1 filter paper). The PCZM content in the filtrate was determined by measuring the absorbance at 254.5 nm after appropriate dilution with methanol. The drug content was determined using the standard calibration curve. The mean percent drug content was calculated as an average of three determinations[15]. For determination of wetting time and water absorption ratio[16], a piece of tissue paper folded twice was placed in a small Petri dish (internal diameter of 5 cm) containing 6 ml of water. A tablet was placed on the paper and the time required for complete wetting was measured. The wetted tablet was then weighed. Water absorption ratio ‘R’ was calculated using the equation: R=100×(W_a_–W_b_)/W_a_, where W_a_ is weight of tablet after water absorption and W_b_ is weight of tablet before water absorption. For determination of *in vitro* dispersion time, one tablet was placed in a beaker containing 10 ml of pH 6.8 phosphate buffer at 37±0.5° and the time required for complete dispersion was determined[17]. IR spectra of PCZM and its formulations were obtained by KBr pellet method using Perkin-Elmer FTIR series (Model 1615) spectrophotometer in order to rule out drug-carrier interactions.

### Dissolution study[18]:

*In vitro* dissolution of PCZM fast disintegrating tablets was studied in USP XXIII type-II dissolution apparatus (Electrolab, Model-TDT 06N) employing a paddle stirrer at 50 rpm using 900 ml of pH 6.8 phosphate buffer at 37±0.5° as dissolution medium. One tablet was used in each test. Aliquots of dissolution medium (5 ml) were withdrawn at specified intervals of time and analyzed for drug content by measuring the absorbance at 255.5 nm. The volume withdrawn at each time interval was replaced with fresh quantity of dissolution medium. Cumulative percent of PCZM released was calculated and plotted against time.

### Stability testing:

Short-term stability studies on the promising formulations (DPM_4_ and DCP_4_) were carried out by storing the tablets in an amber coloured rubber stoppered vial at 40°/75% RH over a 3 mo period. At an interval of 1 mo, the tablets were visually examined for any physical changes, changes in drug content and *in vitro* dispersion time.

## RESULTS AND DISCUSSION

Fast disintegrating tablets of prochlorperazine maleate were prepared by direct compression method employing *Plantago ovata* mucilage and crospovidone as super-disintegrants in different ratios along with microcrystalline cellulose. Directly compressible mannitol (Pearlitol SD 200) was used as a diluent to enhance mouth feel. A total of eight formulations and a control formulation DC_0_ (without super-disintegrant) were designed. As the blends were free flowing (angle of repose <30°, and Carr's index <15%) tablets obtained were of uniform weight (due to uniform die fill), with acceptable variations as per IP specification i.e., below ±7.5%. Drug content was found to be in the range of 95 to 101%, which is within acceptable limits. Hardness of the tablets was found to be about 2.63 kg/cm^2^. Friability below 1% was an indication of good mechanical resistance of the tablets. Water absorption ratio and wetting time, which are important criteria for understanding the capacity of disintegrants to swell in presence of little amount of water were found to be in the range of 50-86% and 11-47 s, respectively. Among all the designed formulations, two formulations, viz., DPM_4_ and DCP_4_ were found to be promising and displayed an *in vitro* dispersion time ranging from 8 to 10 s, which facilitates their faster dispersion in the mouth.

Overall, the formulation DPM_4_ containing 8% w/w of *Plantago ovata* mucilage and 60% w/w of microcrystalline cellulose was found to be promising and has shown an *in vitro* dispersion time of 8 s, wetting time of 11 s and water absorption ratio of 86% when compared to control formulation (DC_0_) which shows 244 s, 247 s and 50% values respectively for the above parameters (Table 2). The experimental data also reveals that the results obtained from the *Plantago ovata* mucilage are comparable and even slightly better than those of crospovidone.

**TABLE 2:** EVALUATION OF FAST DISINTEGRATING TABLETS

Para-meters	Formulation code								
	
	DC_0_	DCP_1_	DCP_2_	DCP_3_	DCP_4_	DPM_1_	DPM_2_	DPM_3_	DPM_4_
Hardness (kg/cm^2^)* ±SD	2.60 ±0.10	2.63 ±0.15	2.63 ±0.15	2.56 ±0.152	2.53 ±0.152	2.63 ±0.05	2.63 ±0.05	2.60 ±0.20	2.63 ±0.05
Thickness (mm)	2.52	2.80	2.92	2.62	2.82	2.78	2.73	2.55	2.74
Friability (%)	0.45	0.40	0.42	0.50	0.48	0.46	0.50	0.52	0.48
*In vitro* dispersion time (s)* ±SD	244.50 ±2.0	46.76 ±2.50	42.50 ±0.58	23.82 ±1.22	10.50 ±0.82	41.50 ±0.89	37.77 ±0.43	18.48 ±0.45	7.86 ±0.72
Wetting time (s)* ±SD	247.9 ±1.62	47.20 ±2.87	45.50 ±1.89	25.99 ±1.59	12.39 ±1.06	44.65 ±0.80	40.14 ±0.77	21.04 ±0.84	11.09 ±0.57
Water-absorption ratio (%)* ±SD	50.00 ±2.78	56.89 ±0.60	63.41 ±1.13	70.37 ±1.00	85.00 ±0.51	63.00 ±0.69	67.17 ±0.14	72.47 ±0.53	85.92 ±0.26
Percent drug content (%)* ±SD	95.68 ±0.59	97.96 ±1.38	99.03 ±0.78	97.76 ±0.73	99.46 ±0.71	99.42 ±1.02	101.27 ±0.74	100.45 ±0.70	100.50 ±0.84
Weight variation (%)	(148-156 mg) within the IP limits of ±7.5%								

* Average of three determinations, formulations DCP_4_ and DPM_4_ were selected as the promising and used in further studies.

**Fig. 1:**
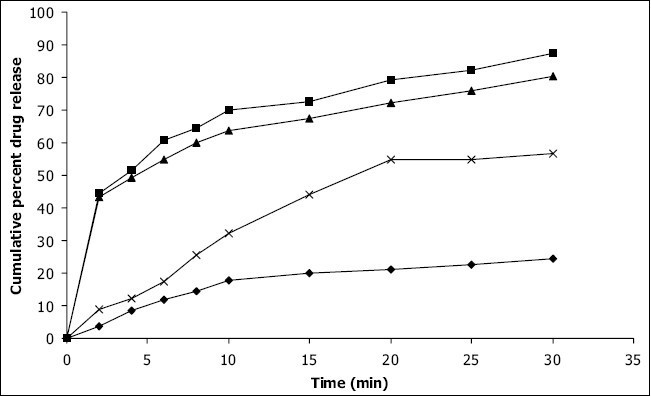
*In vitro* cumulative drug release versus time profiles Plot showing percent cumulative drug release of promising prochlorperazine maleate formulations in pH 6.8 phosphate buffer from control tablet DC_0_ (–◆–); promising DPM4 formulation (–■–); promising DCP_4_ formulation (–▲–); conventional commercial tablet formulation CCF (–x–).

*In vitro* dissolution studies on the promising formulations (DPM_4_ and DCP_4_), the control (DC_0_) and commercial conventional formulations (CCF) were carried out in pH 6.8 phosphate buffer, and the various dissolution parameter values viz., percent drug dissolved in 5 min, 10 min and 15 min (D_5_, D_10_ and D_15_), dissolution efficiency at 10 min (DE_10_ min)[19], t_50%_, t_70%_ and t_90%_ are shown in Table 3 and the dissolution profiles depicted in fig. 1. This data reveals that overall, the formulation DPM_4_ has shown more than five-fold faster drug release (t_50%_ 3.3 min) when compared to the commercial conventional tablet formulations of prochlorperazine maleate (t_50%_ 17.4 min) and released nearly 4-times more drug than the control formulation in 10 min.

**TABLE 3:** *IN VITRO* DISSOLUTION PARAMETERS IN PH 6.8 PHOSPHATE BUFFER

Formulation code	D_5_ (%)	D_10_ (%)	D_15_ (%)	DE_10min_ (%)	t_50%_ (min)	t_70%_ (min)	t_90%_ (min)
DC_0_	10.0	18.0	20.0	26.28	>30	>30	>30
DPM_4_	56.00	70.00	73.00	36.31	3.3	10.00	>30
DCP_4_	52.00	64.00	67.00	37.26	4.10	18.0	>30
CCF	24.00	32.00	44.00	24.75	17.4	>30	>30

DC_0_=Control formulation without superdisintegrant, DCP_4_=formulation containing crospovidone (8% w/w) as superdisintegrant, DPM_4_=formulation containing *Plantago ovata* mucilage (8% w/w) as superdisintegrant, CCF=conventional commercial formulation, D_5_=percent drug released in 5 min, D_10_=percent drug released in 10 min, D_15_=percent drug released in 15 min, DE_10min_=dissolution efficiency in 10 min, t_50%_=time for 50% drug dissolution, t_70%_=time for 70% drug dissolution, t_90%_=time for 90% drug dissolution.

IR spectroscopic studies indicated that the drug is compatible with all the excipients. The IR spectrum of DPM_4_ and DCP_4_ showed all the characteristic peaks of prochlorperazine maleate pure drug, thus confirming that no interaction of drug occurred with the components of the formulation. Short-term stability studies of the above formulations presented in Tables 4 and 5 indicated that there are no significant changes in drug content and *in vitro* dispersion time at the end of 3 mo period (p<0.05).

**TABLE 4:** STABILITY DATA OF DCP_4_ FORMULATION AT 40°C/75% RH

Time in days	Physical changes	Percent drug content±SD*	*In vitro* Dispersion time*
1^st^ day (initial)	--	97.74±0.62	10.50±0.82
30^th^ day (1 mo)	No changes	97.68±0.015	10.60±0.02
60^th^ day (2 mo)	No changes	97.61±0.025	10.95±0.065
90^th^ day (3 mo)	No changes	97.10±0.030	11.55±0.437

* Average of three determinations, Mean per cent drug content on the first day is 97.74±0.62, on day 90 is 97.1±0.03 with a difference between day 90 and the first day being 0.64±0.59

**TABLE 5:** STABILITY DATA OF DPM_4_ FORMULATION AT 40°C/75% RH

Time in days	Physical changes	Percent drug content±SD*	*In vitro* Dispersion time*
1^st^ day (initial)	--	95.68±0.592	7.86±0.72
30^th^ day (1 mo)	No changes	94.91±0.045	7.56±0.60
60^th^ day (2 mo)	No changes	94.84±0.035	7.47±0.19
90^th^ day (3 mo)	No changes	94.75±0.05	7.40±0.12

* Average of three determinations. Mean per cent drug content on the first day was 95.68±0.59, on day 90 was 94.75±0.05 with a difference between day 90 and the first day being 0.98±0.54

## References

[1] Baveja SK, Gupta BM (1968). Rheology of Aqueous dispersions of *Plantago ovata* seed husk-I. Indian J Pharm Sci.

[2] Baveja SK, Gupta BM (1968). Rheology of Aqueous dispersions of *Plantago ovata* seed husk-II. Indian J Pharm Sci.

[3] Mithal BM, Kasid JL (1964). Evaluation of emulsifying properties of *Plantago ovata* (Ispaghula) seed husk. Indian J Pharm Sci.

[4] Mithal BM, Kasid JL (1965). Evaluation of the suspending properties of Plantago ovata (Ispaghula) seed husk. Indian J Pharm Sci.

[5] Kulkarni GT, Gowthamarajan K, Rao BG, Suresh B (2002). Evaluation of binding property of *Plantago ovata* and *Trigonella Foenum gracecum* mucilage. Indian Drugs.

[6] Washi SP, Sharma VD, Jain VK, Sinha P (1985). *Plantago ovata*: genetic diversity, cultivation, utilization and chemistry. Indian J Nat Prod.

[7] Seager H (1998). Drug delivery products and the Zydis Fast Dissolving Dosage Forms. J Pharm Pharmacol.

[8] Chang RK, Guo X, Burnside BA, Cough RA (2000). Fast dissolving tablets. Pharm Tech.

[9] Dobetti L (2001). Fast-melting tablets: Developments and Technologies. Pharma Tech.

[10] Kuchekar BS, Arumugam V (2001). Fast Dissolving Tablets. Indian J Pharm Educ.

[11] Sweetman SC (2002). Martindale: The Complete Drug Reference.

[12] Baveja SK, Rao KV, Arora J (1989). Examination of natural gums and mucilages as sustaining materials in tablet dosage forms-II. Indian J Pharm Sci.

[13] Kuchekar BS, Badhan AC, Mahajan HS (2004). Mouth dissolving tablets of salbutamol sulphate: A novel drug delivery system. Indian Drugs.

[14] Banker GS, Anderson GR, Lachman L, Liberman HA, Kanig JL (1987). Tablets. The theory and practice of industrial pharmacy.

[15] (1996). Indian Pharmacopoeia.

[16] Chaudhari PD, Chaudhari SP, Kohle SR, Dave KV, More DM (2005). Formulation and evaluation of fast dissolving tablets of famotidine. Indian Drugs.

[17] Bi YX, Sunada H, Yonezawa Y, Danjo K (1999). Evaluation of rapidly disintegrating tablets by direct compression method. Drug Develop Ind Pharm.

[18] Bhagwati ST, Hiremath SN, Sreenivas SA (2005). Comparative evaluation of disintegrants by formulating cefixime dispersible tablets. Indian J Pharm Educ Res.

[19] Khan KA (1975). The concept of dissolution efficiency. J Pharm Pharmacol.

